# 3D Printing: Applications in evolution and ecology

**DOI:** 10.1002/ece3.5050

**Published:** 2019-03-13

**Authors:** Matthew Walker, Stuart Humphries

**Affiliations:** ^1^ School of Life Sciences University of Lincoln Lincoln UK

**Keywords:** 3D printing, ecology, evolution, experimental design, methods

## Abstract

In the commercial and medical sectors, 3D printing is delivering on its promise to enable a revolution. However, in the fields of Ecology and Evolution we are only on the brink of embracing the advantages that 3D printing can offer. Here we discuss examples where the process has enabled researchers to develop new techniques, work with novel species, and to enhance the impact of outreach activities. Our aim is to showcase the potential that 3D printing offers in terms of improved experimental techniques, greater flexibility, reduced costs and promoting open science, while also discussing its limitations. By taking a general overview of studies using the technique from fields across the broad range of Ecology and Evolution, we show the flexibility of 3D printing technology and aim to inspire the next generation of discoveries.

## 1 INTRODUCTION

Studies of ecology and evolution often use equipment which has been made by the investigators, for a diverse range of applications and exhibits a high level of creativity. 3D printing offers an opportunity to produce equipment which can be shared with the scientific community, allowing other investigators to replicate studies with more accuracy than before, strengthening the open science movement (Baden et al., 2015; Pearce, 2014). Furthermore, 3D printing offers timesavings over traditional manufacturing methods, as the process is largely automated. As well as simple models, 3D printing makes it possible to generate complex morphologies accurately, produce laboratory equipment and generate models for teaching and outreach. 3D printing also removes ethical concerns over using live animals for experimental manipulation.

Previous studies that have used models in one form or another could be improved using 3D printing (e.g., in Fraisse, Bormans, & Lagadeuc, 2015). Many studies might simply benefit from the increased consistency of the technique, reduction in costs, and ease of production. For some studies, 3D printing would offer the opportunity to conduct the research using more life‐like models, which may elicit more appropriate responses from the organisms involved.

**Figure 1:**
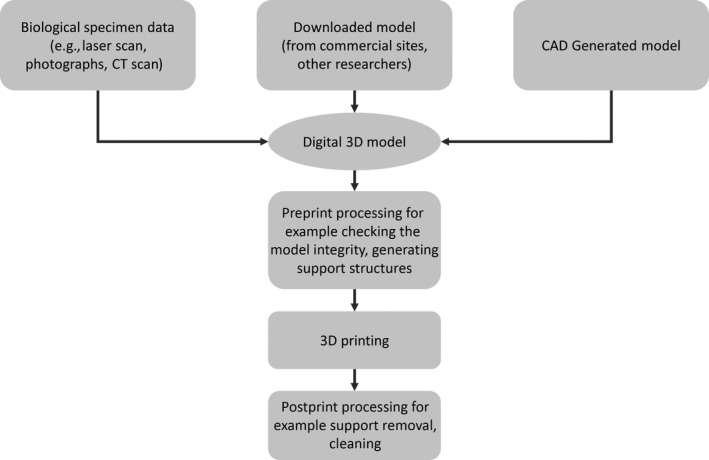
A generalized work flow of 3D printing. The digital model can be obtained from a biological specimen. If the model is laboratory equipment or does not need to be an exact replica of the structure of interest (e.g., Campos et al., 2015, see below) then the model can be generated using Computer Aided Design (CAD). Finally, either type of model (i.e., CAD or scan based) can be downloaded from commercial sites, collaborators, or other researcher's publications

The technique of 3D printing encompasses a range of manufacturing methods (see Table 1, Box 1 for an introduction and Figure 1 for a generalized workflow), which may be referred to as rapid prototyping, desktop‐, additive‐, or rapid‐manufacturing. Originating with stereolithography (SLA) using a specific photosensitive polymer (Wong & Hernandez, 2012), the technology now allows printing with a variety of materials with diverse properties (Berman, 2012, see Table 1), including soft, flexible materials (Abdollahi, Davis, Miller, & Feinberg, 2018). Each 3D printing method has its own advantages and disadvantages (see Table 1), but overall the technique offers a method of creating objects in a way that is more like organic growth than traditional “subtractive” methods. Rather than removing material to create an object, 3D printing builds layers up by adding material as a series of thin slices (Wong & Hernandez, 2012).

**Table 1:** A brief overview of 3D printing methods

Starting material		Method	Layer thickness (µm)	Method overview	Advantages	Disadvantages	Example of use in Ecology and Evolution
Solid	Plastic filament	Fused Filament Deposition (FFD)	300–1,000	Plastic filament is melted and placed in layers by the print head	Common plastics, such as ABS, can be used. Chemical properties retained	Anisotropic (strength differs in Z‐axis, to X‐Y axis). Stepped surface, which lacks fine details	Thermal ecology model lizards (Watson & Francis, 2015)
Powder	Metal	Laser melting	20–100	Thin layers of powder are spread over a bed which the laser selectively melts. Once solidified, a new layer of powder is spread	>99% of the metal's density can be achieved, therefore good mechanical properties	Expensive and slow, limited metals can be used, it is not suitable for reactive metals	
		Electron beam	0.3–100	Powdered metals are selectively melted by electron beam in a vacuum	As for Laser melting. Reactive metals (e.g., Titanium) can be used	Expensive, slow and limited metals can be used	
	Plastic Metal	Laser sintering	100–300	The laser selectively melts powdered plastics and the model is made in the powder bed	Common plastics can be used. Cheap for small number of objects. Chemical properties of material are retained	Poor surface finishes and tolerances are limited	Ceramic filter holder (Lücking et al., 2015)
	Any powder	Binder jetting	90–200	Powdered material is placed in layers over the build area and then glued together with a binding agent	Any powdered material can be used. Relatively fast and cheap. Color models are possible	Produces fragile parts which need further treatment. Poor surface finish	
Liquid	Photo‐polymer	Stereolithography (SLA)	16–150	A laser selectively solidifies thin layers of photopolymer. The print bed moves down to allow a new layer of polymer to form	Accurate with good surface finish that can capture fine details	Expensive and photopolymers are not stable long‐term	Model Neotropical bush‐cricket (Jonsson et al., 2017)
		Photopolymer jetting	16–500	Thin layers of photopolymer are jetted on to the build area. They are cured with ultraviolet light straight away	Multiple materials can be used. Can be high precision	Expensive and photopolymers are not stable long‐term	Models of black widow spiders (Brandley et al., 2016) Mold for microfluidic devices (Kamei et al., 2015)
	Wax	Material jetting	13–50	Inkjet printer heads drop hot wax on a bed, which cools and forms layers	Accurate with good surface finish. Suitable for lost wax casting	Slow process that produces fragile models	Replicating delicate dinosaur bones (Bristowe et al., 2004)

Examples, from papers cited in the main text are provided to illustrate applications. Layer thicknesses are approximate as specific makes and models of 3D printers will vary. A guide of prices for different technology types can be found in Table 2

Box 1 **3D printing: an introduction**

1 Before 3D printing an object, the technique to be used needs to be chosen. Table 1 outlines some 3D printing methods and Table 2 outlines some costs and features of 3D printers. Important considerations include the layer thickness; whether, if the object has cavities; and the minimum feature size required. For example, FFD 3D printers can print objects with cavities, but produce relatively thick layers and so the model will likely have a stepped appearance, especially on curved surfaces. SLA 3D printers have thinner layer thickness so curved surfaces are smoother, but they cannot create cavities.

The first step of 3D printing is obtaining a digital model, this can be based on CT‐scan, laser scan data, or it could be designed using a computer (Computer Aided Design, CAD). This model is digitally sectioned into thin layers, by the 3D printer software and these layers dictate the printer head's path. In the case of SLA, the “head” is a laser while for FFD it is a nozzle extruding molten plastic filament.

Once the 3D printer has produced the model, there is often a level of postprint processing. The model may need to be cleaned in alcohol to remove excess resin in the case of SLA. Most 3D printers need a scaffold of material to support the model during printing, which needs to be removed after printing. In some cases, the support material is different from the final model (e.g., FFD) so can be dissolved, otherwise it needs to be cut (e.g., SLA). Powder based 3D printing technologies use the surrounding powder to support the model during printing. This means that the model will need to be removed and dusted, while some powder‐based models may need impregnating with binder (glue or similar) to solidify the object completely.

**Table 2:** A range of different 3D printers, their cost (as of February 2019) and features

Technology	Type	Manufacturer	Printer	Cost (USD)	Build volume (mm)	Maximum resolution (µm)	Connectivity
FFD	Desktop	Prusa Research	Original Prusa i3 MK3	$999	250 × 210 × 200	50	USB
	DIY/kit	Creality	Ender 5	$329	220 × 220 × 300	50	USB, SD card
	Desktop	Sindoh	3DWOX 1	$1,499	210 × 200 × 195	50	USB, Ethernet, Wi‐Fi
	Desktop	Qidi Tech	X‐ONE 2	$279	140 × 140 × 140	50	NS
SLA	Desktop	ELEGOO	Mars	$349	120 × 68 × 155	50	USB
	Desktop	Photon	ANYCUBIC	$519	115 × 65 × 155	30	USB, SD card
	Professional	B9Creations	B9Creator v1.2	$4,595	104 × 75 × 203	30	USB
	Professional	Formlabs	Form 2	$3,499	145 × 145 × 175	30	USB, Ethernet, Wi‐Fi
Binder Jetting	Industrial	3D systems	ProJet CJP 660Pro	$50,000‐$100,000	254 × 381 × 203	10	Ethernet, Wi‐Fi
Material Jetting	Industrial	Stratasys	Object Eden260VS	$50,000‐$100,000	255 × 252 × 200	20	Ethernet
	Industrial	3D systems	ProJet 3500HD MAX	$50,000‐$100,000	298 × 185 × 203	20	Ethernet, Wi‐Fi
Powder binding	Industrial	EOS	P396	>$250,000	340 × 340 × 600	60	Ethernet
	Industrial	EOS	Formiga P110	$100,000‐$250,000	200 × 250 × 330	60	Ethernet

Notes

NS: not stated.

Data from www.aniwaa.com, 3D printers with the highest customer rating for each technology were chosen.

In many fields, 3D printing is an established methodology. In medicine, it has been used for almost 20 years to make surgical guides used to plan surgery (Cohen, Laviv, Berman, Nashef, & Abu‐Tair, 2009; D'Urso, 1999; Gerstle, Ibrahim, Kim, Lee, & Lin, 2014; Rengier et al., 2010). Technological developments now allow 3D printing of metals (Murr et al., 2012), permitting prosthetics to be custom‐made for the patient (Sing, An, Yeong, & Wiria, 2016). 3D printing is used in microbiology (Connell, Ritschdorff, Whiteley, & Shear, 2013), tissue culture, and the development of replacement (Duan, Hockaday, Kang, & Butcher, 2013), and bionic (Mannoor et al., 2013) organs. In all of these areas, the technique has led to the development of new methods (Cohen et al., 2009; Connell et al., 2013; D'Urso et al., 1999; Duan et al., 2013; Ebert et al., 2009; Herbert, Simpson, Spence, & Ion, 2005; Mannoor et al., 2013; Rengier et al., 2010). With falling costs (Hoy, 2013), 3D printing has also become more common in Science and Engineering and, its use is rapidly expanding.

There are also many instances where standard equipment does not meet the needs of the investigators (Lücking, Sambale, Beutel, & Scheper, 2015). However, with some knowledge of CAD software, many bespoke items can be designed and printed in‐house. Furthermore, the files can be easily sent to collaborators or included with publications. For example, microfluidic devices are commonly used for a range of experiments involving processes such as bacterial chemotaxis (Ahmed, Shimizu, & Stocker, 2010), pollution monitoring, clinical diagnosis, drug discovery, and biohazard detection (Holmes & Gawad, 2010; Yazdi et al., 2016). Where printing resolution allows, the use of 3D printing to create either molds for Polydimethylsiloxane (PDMS) chambers or to directly print the chambers is becoming increasingly popular (Bonyár et al., 2010; Ho, Ng, Li, & Yoon, 2015; Kamei et al., 2015; Kitson, Rosnes, Sans, Dragone, & Cronin, 2012; Lee et al., 2014; Yazdi et al., 2016) over the time‐consuming and often expensive process of creating a mold using traditional techniques (Waldbaur, Rapp, Länge, & Rapp, 2011). The ability to quickly produce and test designs using the 3D printing process is also highly valuable, especially where new devices need to be developed.

3D printing can also significantly reduce the cost of standard laboratory equipment (e.g., laboratory jacks, retort stands, Eppendorf pipettes, and equipment holders), by up to 97% compared to vendors pricing (Zhang, Anzalone, Faria, & Pearce, 2013). Despite the high initial cost of a 3D printer, the cost per unit in materials is low (Kitson et al., 2012; Waldbaur et al., 2011). In addition to financial benefits, time savings can be dramatic. Electronic files containing all the information needed to 3D print equipment can be obtained from a number of online sources (Baden et al., 2015; Willermet, 2016, as used by Brandley, Johnson, & Johnsen, 2016) and printed immediately. Thus, even with next day shipping from equipment vendors, downloading and 3D printing is far faster (Zhang et al., 2013). As hardware reduces in cost 3D printing becomes accessible to individuals and small laboratory groups (Hoy, 2013). Additionally, there are now a number of commercial print‐on‐demand 3D printing companies, allowing researchers with more limited budgets to obtain printed models without the cost of buying hardware (e.g., McDougal & Shepherd, 2015).

Another blossoming application is the use of 3D models in teaching and learning, a key part of encouraging the next generation of scientists and engaging the public with research. Students with visual impairment, and members of the public can explore both large (Larkin, personal communication) and microscopic fossils (Kaplan & Pyayt, 2015; Rahman, Adcock, & Garwood, 2012; Teshima et al., 2010) which can be scaled to be easily held. This approach has been used to enable those with visual impairment to learn about microscopic planktonic organisms (Teshima et al., 2010), as well as cells undergoing mitosis, striated muscle cells, and neuromuscular junctions (Kolitsky, 2014). Such models could also be used by students who are unable to use microscopes due to motor impairment. Furthermore, to make complex microscopic features accessible bacteria, viruses, proteins, enzymes (Gardner & Olson, 2016), molecules (Bara, Heath Turner, Zhang, & Hawkins, 2015), Natural Killer cells (from high‐resolution micrographs, Mace, Moon, & Orange, 2015), and neurons (McDougal & Shepherd, 2015) have all been 3D printed. Compared to digital models and textbooks, models of complex anatomical structures, such as the human heart (Kaplan & Pyayt, 2015) and horses’ feet (Preece, Williams, Lam, & Weller, 2013), provide the opportunity for the learner to interact with the subject, permitting physical exploration of the structures.

This review focuses on the advances within Ecology and Evolution, and related fields, which have been driven by 3D printing. Examples of how 3D printing could be used are provided throughout, with the hope of inspiring the reader.

## 2 EVOLUTION

### 2.1 Morphology and coloration

Understanding both the inter‐ and intra‐specific signals organisms convey to one another is a core aim for evolutionary, ecological, and behavioral studies. Unpicking relationships between organisms and the signals that mediate them is often achieved through manipulative experiments (Andersson, 1994; Krebs & Davies, 1993). Here, 3D printed objects offer great benefit as they can be produced to specifications not possible with real organisms. An illustrative example is animal coloration. The communication of an individual's fitness through coloration is common, especially in males (Andersson, 1994; Krebs & Davies, 1993; Svensson & Wong, 2011). Testing the influence of coloration on mating success or other interactions is normally done through comparison between an individual's brightness and the rest of the population (Crothers & Cummings, 2015). However, this process is time consuming and may require the capture, and associated stress, of animals. Being able to produce 3D printed models of animals, that can be colored to account for the species’ visual system, allows direct manipulation of a visual signal, while maintaining (and controlling) morphology to encourage responses. This approach has been used to investigate the coloration of the poison frog*, Oophaga pumilio*, where brightness was found to be an indicator of more aggressive males (Crothers & Cummings, 2015). Similarly, Brandley et al. (2016) investigated the evolution of the red “hourglass” mark of the black widow spider (*Latrodectus* spp.), which is believed to be aposematic (a warning to deter predators). Hand‐painted 3D printed models of the spiders received fewer predation attempts when models exhibited the red hourglass marking (Brandley et al., 2016).

While bright coloration is often considered an honest sexual signal, the presence of distinctive colors may result in high predation rates, reduced immune response and increased oxidative stress (Svensson & Wong, 2011), suggesting a trade‐off between natural and sexual selection. For example, individuals in low‐risk environments are often more brightly colored than those in high‐risk environments (Endler, 1982). Heinen‐Kay et al. (2015) used 3D printing to investigate risk‐moderated coloration in the Bahamas Mosquitofish (*Gambusia hubbsi*). In blue holes (large landlocked sinkholes) lacking predatory fish, male *G. hubbsi* have evolved a brighter orange coloration on their dorsal fin, compared to those in blue holes with predators. On the face of it, the simplest method to investigate potential trade‐offs would have been to translocate fish between blue holes, to observe the effect of coloration on mating success and predation. However, this is not only logistically challenging, but also ethically questionable. By using 3D printed models of males with and without coloration, the potential trade‐off could be easily examined, and results indicated that signal diversity can be driven by the interaction of natural and sexual selection (Heinen‐Kay et al., 2015).

Another example is the coloration of brood‐parasite eggs. Igic et al., (2015) used 3D printing to separate the effects of coloration on the rejection of brown‐headed cowbird (*Molothrus ater*) eggs, by American robins (*Turdus migratius*). Igic et al. (2015) demonstrate how 3D printing can be used to remove human error when reproducing models that would otherwise have to be made by hand, while also allowing the flexibility for each batch of eggs to have a unique shape.

**Figure 2:**
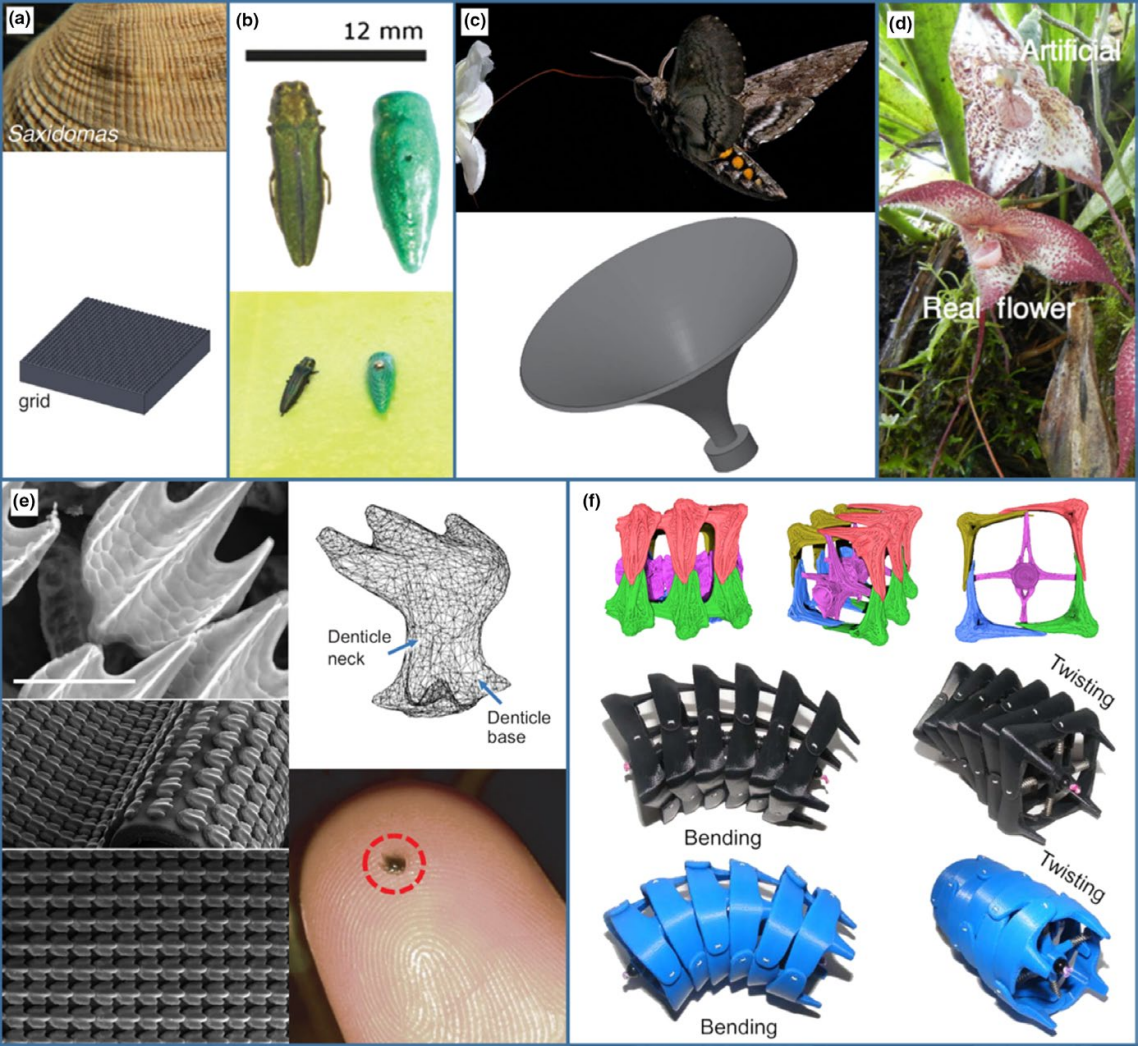
Examples of 3D printed objects from the studies described in the text. (a) Top, Butterclam shell (*Saxidomas nuttalli*), and below textured 3D print replicating surface structure to test ice formation (Mehrabani et al., 2014). Reproduced with permissions from PeerJ. (b) Left, a dead female Emerald Ash Borer (*Agrilus planipennis*) decoy used to bait traps and on the right a 3D printed model. Below, the real decoy and model mounted on a trap (Domingue et al. 2015). Reproduced with permissions from Journal of Pest Science. (c) Top, a hawkmoth (*Manduca sexta*), using its proboscis to probe a flower, and below a CAD model of the 3D printed “flowers” used by Campos et al. (2015). Reproduced with permissions from Functional Ecology. (d) Flower of the Dracula Orchid (*Dracula lafleuri*) and silicone model used to separate visual and olfactory cues (Policha et al., 2016). Reproduced with permissions from New Phytologist. (e) Top left, an Environmental Scanning Electron Microscope image of bonnethead shark (*Sphyrna tiburo*) skin. Scale bar 100 µm. Top right, a digital model of a denticle. Bottom left, SEM images of 3D printed denticles embedded in membrane. Bottom Right, a single 3D printed denticle approximately 1.5 mm in length (Wen et al., 2014). Reproduced with permissions from Journal of Experimental Biology. (f) Top, Micro‐CT scan images of a seahorse (*Hippocampus* spp.) tail. Middle, a 3D printed model based on the micro‐CT scan. Bottom, a 3D printed hypothetical tail structure. These models were used to assess how the square cross‐section of the seahorse tail grips and resists crushing compared to the round cross‐section (Porter et al., 2015). Reproduced with permissions from Science

3D prints do not always need to be the final model—they can also be used to create a mold. This approach was used by Policha et al. (2016) to understand the visual and olfactory components of flower attraction. Silicone models from 3D printed molds allowed the separation of visual and olfactory cues in the Dracula orchid (*Dracula lafleuri*). This species mimics mushrooms to attract flies, which then pollinate the plant. The authors used accurate scent‐free silicone models (Figure 2d) to show that flies are attracted to both visual and olfactory cues, with a synergistic effect suggesting that it is driven by multimodal mimicry of the mushrooms by the plant (Policha et al., 2016). Similarly, Campos et al. (Campos, Bradshaw, & Daniel, 2015) used 3D printed flower analogues to investigate the effect of flower morphology on hawkmoth (*Manduca sexta*) feeding. Using flower mimics from a flat disk to a realistic trumpet shape (see Figure 2c), they found support for the theory that flower trumpets act as a mechanical guide, despite the printed “flowers” lacking the coloration and flexural properties of the real flower (Campos et al., 2015). Both models produced for these studies would be difficult to fashion using traditional techniques, and the studies show how CAD and 3D printing allow flexibility in design while facilitating the production of multiple identical units.

### 2.2 Biomechanics

Biomechanics allows researchers to link form to function, a key part of evolutionary biology. Here, 3D printing offers an opportunity to produce realistic models of whole or parts of organisms which can be used to test theories about shape and function of structures. As demonstrated by Policha et al. (2016) with the Dracula orchid, exploring organismal function with models using a reductionist approach, that is examining component parts, is often useful. Porter, Adriaens, Hatton, Meyers, and McKittrick (2015) produced 3D printed models to investigate the functional morphology of the seahorse (*Hippocampus *spp.) tail. Seahorses have a square cross‐section tail, as opposed to the round cross‐section tails which might be expected of fish. Hypothetical round cross‐section tails were generated using CAD and compared to the square morphology of seahorse tails, which was obtained from CT scan data. To compare possible functional advantages of square cross‐section tails Porter et al. (2015) then 3D printed both tail morphologies and subjected them to a series of tests. The authors found that square cross‐section prototype tails (Figure 2f) performed better for grasping and are more resistant to crushing than round‐section tails. Improved grasping ability of the square cross‐section tail was likely an evolutionary advantage for seahorses, which use their tails to hold onto corals, algae, and seagrasses (Neutens, de Dobbelaer, Claes, & Adriaens, 2017; Porter et al., 2015). By examining the tail in this way, Porter et al.'s work helps to explain some of the unusual morphology that seahorses have evolved.

In the Neotropical buch‐cricket (*Acanthacara acuta*), the sound produced by the wings is amplified by an unusual extension of the pronotum (the dorsal covering of the thorax), which forms a chamber over the wings. To explore the function of this chamber, 3D printed models have been used to replicate the chamber (Jonsson et al. 2017), which is hypothesized to work as a Helmholtz resonator (Morris & Mason, 1995). By changing the material properties of the chamber (photopolymer resin in the 3D model, instead of insect cuticle), Jonsson et al. (2017) showed that the morphology of the structure alone is responsible for the amplification of sound. Surface texture can also be modeled well by 3D printing. In an examination of ice formation on surfaces, 3D printing was used to replicate surface textures of blue mussels, (*Mytilus edulis*), Antarctic sea urchin (*Sterechinus neumayeri*), and sub‐polar butterclams (*Saxidomas nuttalli*) (Figure 2a). MehrabaniRay, Tse, and Evangelista (2014) found that the ridges and bumps present on all tested surfaces reduced ice formation at −20°C, but the role of surface texture was limited to approximately 6%. Such 3D models have the advantage of having the same material properties, allowing only the variable under investigation to be modified.

Another use of 3D printing is to produce low cost, highly accurate objects for calibration. Many studies use cameras to record and facilitate later analysis, and cameras must be calibrated beforehand for measurement data to be meaningful. Koehler, Liang, Gaston, Wan, and Dong (2012) investigated deformation of dragonfly wings during flight to better understand the aerodynamics, structural dynamics, and control of the wing. By 3D printing their calibration rig (with a resolution of 25 µm), the calibration points could be placed accurately, permitting the exacting calibration required for this study.

### 2.3 Fluid dynamics

Biological fluid dynamics is an area that is being changed dramatically with the use of 3D printing. The complex geometries of sponges and corals have been 3D printed to enable investigations of the fluid flow around organisms without the need to culture or remove them from their habitats (Kruszyński & van Liere, 2009). 3D printed models could also be used to examine the flow of fluids through organisms, such as the machined steel models of sponges used by Vogel (1974), by offering more realistic and easily modified 3D models. Complex internal structures, for example sponge canals, or vertebrate nasal passages, can be replicated in clear plastics (or resins) for visualization, and Particle Image Velocimetry (PIV, Stamhuis, 2006) can be used to quantify fluid flows to provide an understanding of form and function for both external and internal structures. In other instances, the size of an organism may present difficulties, both for the production of life‐sized models and conducting research. Padisák, Soróczki‐Pintér, & Rezner, (2003) used PVC‐U and modeling material to create scaled‐up models of microscopic planktonic organisms (real size 40–200 µm, model sizes 5–35 cm) to investigate the effect of morphology on sinking rate. These construction methods resulted in greatly simplified models, much like Furbish and Arnold's (1997) use of beeswax and pins to produce models (2–6 cm) of foraminifera (real size ~150–1200 µm). As suggested by Fraisse et al. (2015), studies of microscopic plankton could be improved by using 3D printed, biologically realistic models, based on highly accurate CT or *SEM* images.

As well as enabling production of scaled models of microscopic structures, 3D printing can be used to recreate small structures while providing the opportunity to manipulate their shape. Shark skin has small tooth‐like projections (denticles), which have been studied since the 1970s for applications in industry, as they are thought to reduce drag and have antifouling properties (Oeffner & Lauder, 2012; Pu, Li, & Huang, 2016). Wen, Weaver, and Lauder (2014) used 3D printed shark skin (Figure 2e) to examine water flow over denticles, finding that they reduce drag compared to a flat surface, leading to a predicted increase in swimming efficiency under certain conditions. In this instance, 3D printing allowed manipulation of the surface (i.e., denticle distribution and flexibility of the “skin”) and removed ethical issues of using real skin. Wen et al.'s (2014) findings were echoed by Lauder et al. (2016), who also demonstrated the ability of 3D printing to accurately produce objects which are microscopic (10 by 15 µm).

3D printed models can be used to test or ground‐truth computational models and to perform experiments which would not be otherwise possible. An example is the recreation of the airways of *Scincus scincus*. This species of skink spends its life below the sand, moving in a fish‐like manner, hence its common name “sandfish lizard.” Despite breathing while under the sand, Stadler et al. (2016) found no evidence of sand inside dissected sections of the animals’ airways. They theorized that the airways had morphological adaptations, possessing aerodynamic properties which limited sand ingress. To test this, Stadler et al. (2016) used computational and 3D printed models. As the airways are small delicate structures they scaled the airways up (changing the working fluid to maintain the ratios of forces acting on the model) and used larger particles of sand. While the 3D printed models were not able to fully recreate the inhalation and exhalation velocities seen in vivo, 50% of the tests resulted in no sand being present inside of the model airways, lending support to the computational models.

As evidenced above, 3D printing can be used to produce models that would simply not be possible using other methods. As we have seen 3D printing allows the production highly complex and detailed models that can be used for research. Printed models of extinct organisms, for example, can be used to measure efficiency of flight or swimming, helping us to understand behavior and function of prehistoric creatures (e.g., swimming methods of plesiosaurs, Muscutt et al., 2017).

## 3 PALEOBIOLOGY AND CURATION

Reproducing fossil material is traditionally achieved using molds and casts (Waters, 1983), which often involves high temperatures (Benton & Walker, 1981) or chemicals (Purnell, 2003; Spjeldnaes, 1963), which can be harmful and may damage delicate specimens (Bristowe, Parrott, Hack, Pencharz, & Raath, 2004; Purnell, 2003). In comparison, scanning and 3D printing of fossil material has far fewer risks. In an early example of 3D printing in this field Bristowe et al. (2004) created models of the thin bones of the dinosaur *Coelophysis rhodesiensis* to avoid damaging the fragile fossil. However, as the technology available at the time was limited to Fused Filament Deposition (FFD) 3D printing (Table 1) with paraffin wax, the prints were easily damaged. Such delicate paraffin wax bones could now be printed using alternative methods and materials to produce more robust models. In some instances, scanning and 3D printing has allowed nondestructive recreation of remains that only exist as cavities in a stone matrix, such as Clark's , Adams, Lawton, Cruickshank, and Woods (2004) print of the negative space of a cavity to recreate the skull of a mammal‐like reptile (dicynodont). Without Magnetic Resonance Imaging (MRI), there would be no way to visualize the complex skull from the inaccessible cavity, but from this scan 3D printing offers an opportunity to have a physical replica which can be held and observed.

Mitsopoulou et al. (2015) used computational models and statistical methods to recreate missing bones from an incomplete skeleton of the extinct dwarf elephant (*Palaeoloxodon tiliensis*) using 3D printing. Another example is the Lapedo child's skull, which was broken into many pieces and had undergone taphonomic distortion in the 24,500 years since its burial (Almeida et al., 2007). The skull was digitally reconstructed then 3D printed twice; one of the skulls was placed on display and the other used for facial reconstruction (Almeida et al., 2007).

In the past, models have been a major point of interaction with both the public in museums and for teaching. These models are often expensive and can be fragile. 3D printed models are often robust and can be made in‐house relatively quickly and cheaply. These models can be printed in color, and dependent on the method a range of colors can be offered and prints can include multiple colors and transparent material (Begolo, Zhukov, Selck, Li, & Ismagilov, 2014; Sitthi‐amorn et al., 2015), so that internal details can be seen (Blackburn, 2017). These accurate models can be interactive, such as a model of a flint axe (Galvin, 2017), allowing people to understand more about an object (in this case how it was constructed).

Niven, Steele, Finke, Gernat, and Hublin (2009) envisaged the use of 3D printing as an opportunity for museum collections to expand the number of exhibits they hold by repairing skeletons, or by combining pieces of specimens already present in collections with 3D printed pieces. For example, the Quagga (*Equus quagga quagga*) skeleton on display in the Grant Museum of Zoology (University College London) has a 3D printed left leg replacing a missing limb, created by CT scanning the right leg and mirroring the data (Larkin & Porro, 2016). This approach is also being used to replace forelimb bones missing from a recently deceased Fin Whale, *Balaenoptera physalus* (Larkin, personal communication). By using a photogrammetric scan of the forelimb of a different specimen it has been possible to appropriately scale and print replacement bones for mounting with the real skeleton (Larkin, personal communication). Alternatively, scanned animals from other collections might be printed in a museum so that more locations have copies of specimens. By diversifying their natural history collections, museums can facilitate research and allow visitors to experience more (Niven et al., 2009).

Using 3D printed models reduces damage to fossils by: not requiring climate (temperature and humidity) controlled display cases making them both easier and cheaper to display (Almeida et al., 2007); the fossils do not have to be transported to and from mount‐maker's studios; and 3D prints prevent over handling of the fossils, while allowing the creation of custom well‐fitting support structures (Mallison, 2011). Instead of mounting the fossil material, lighter 3D printed replicas can be used, which require fewer mounts. As the 3D print can be drilled into, more esthetically pleasing internal support structures can be built. 3D printing can also allow replicas of scientifically important but normally inaccessible parts of specimens such as the palate or inner ear bones to be placed on display (Larkin, personal communication) and for use in research.

As Koehl (2003) suggests, it is often quicker to make a physical model than to develop a computer simulation. The process of making physical models can now be even more accurate, as 3D printing based on CT scans allows increased realism, particularly as the use of models in research has traditionally been something of an art form. For example, physical modeling has been used in the assessment of how suitable feathers seen on preserved fossils of dinosaurs (*Microraptor gui*) could have been suitable for gliding or flight (Koehl, Evangelista, & Yang, 2011). The models of *M. gui* used were originally made from foam with a wire skeleton, then updated to a steel and aluminum skeleton with polymer clay “flesh,” with feathers inserted to the foam or clay. By using 3D printing to create accurate skeletons it should be possible to build more realistic models that allow for more natural placement of feathers.

## 4 ECOLOGY

Models of organisms or their parts are an excellent way to disentangle covarying factors (Koehl, 2003). In some instances, 3D‐printed models need not be biologically accurate. This has been exploited, for example, to isolate the influence of shape, color, odor, and chemical rewards in plant pollination. While flower nectar is an attractant and reward (Thomson, Draguleasa, & Tan, 2015), some nectar also contains caffeine, which both a stimulant and toxic to most organisms. While flower nectar is an attractant and reward, many plants produce nectar that is toxic or repellent to some floral visitors (Adler, 2000). Investigating this counterintuitive pairing in nature had proved almost impossible and previous studies reported mixed results. However, Thomson et al. (2015) used 3D prints to mimic the function of a flower's anther and stamen in collecting and depositing pollen. They printed small hoppers that deposited dye onto a bee when it brushed under the hopper to reach the nectar. By adding sticky tape, the hopper could also collect previously deposited dyes from the bees. Honeybees (*Bombus impatiens*) were presented with jars containing nectar with different caffeine concentrations. By measuring the amount of dye transferred, the authors found that the higher the caffeine content of the nectar the more bee visits (Thomson et al., 2015), possibly due to the improvement in memory formation provided by caffeine (Wright et al., 2013).

### 4.1 Conservation and monitoring

As we have shown, realistic models can be used in many areas of research, but they also have great potential for management and conservation. Current methods of capturing Emerald Ash Borers (*Agrilus planipennis*), an invasive species in North America, commonly use sticky traps baited with a dead female to attract males (Domingue et al., 2015). To produce the bait, females must be caught, killed, and mounted with pins. Simplified 3D printed models of a female Ash Borer (Figure 2b) have now been successfully used to bait traps, and have the advantage of being cheaper, longer lasting, and are able to be mass‐produced (Domingue et al., 2015). While work is still needed to look at the larger‐scale efficacy of these traps, we can easily envisage the use of similar models to attract animals, for instance, to camera traps as a method of monitoring populations.

Watson and Francis (2015) trialled the use of 3D printed ABS (Acrylonitrile Butadiene Styrene), a commonly used plastic, to produce models for studies of thermal ecology. Models are used to establish the distribution of environmental temperatures experienced by an organism, if it experiences no thermal regulation. Copper models are often used for this, but they are often poorly detailed, and are time consuming and difficult to construct. Copper models are produced using a paraffin wax mold of the animal, which is then electroplated, and the wax melted and drained. Copper tubing is often substituted for a detailed model but makes a poor substitute (Bakken & Angilletta, 2014). With reduced costs per model (albeit initial setup is more expensive), higher biological accuracy, reduced production times (1.55 hr compared to 29.83 hr for copper models) and robust nature of the models, Watson and Francis (2015) suggest that 3D printed models for thermal studies have considerable advantages over traditional copper models, especially as the performance of both models is equal.

As of 2016, there has been a population explosion of ravens (*Corvus corax*) in the Mojave Desert. The increased numbers of predatory ravens are negatively impacting the populations of newly hatched Desert tortoises (*Gopherus agassizii*), a vulnerable species (IUCN, 2016). 3D‐printed model tortoises that emit aerosol irritants when attacked are being used to condition the ravens not to eat the tortoises, thereby reducing predation (Shields, Personal Communication), this is a ground‐breaking use of 3D printing in a conservation effort. 3D printing has also been used to help recreate coral reefs. The Great Barrier Reef is experiencing widespread coral bleaching and coral death (Wolff, Mumby, Devlin, & Anthony, 2018), but advances in 3D printing are enabling the production of coral shaped objects, 1 m in height (Sustainable Oceans International, 2012). These large‐scale 3D printed forms replicate the complexity of natural coral, providing organisms such as fish with suitable habitats while corals colonize the external surfaces.

In addition to producing items used directly in research or for teaching, 3D printing can also be used to create mock‐ups of expensive or delicate equipment to test positioning or attachment methods. Chan et al. (2016), used 3D printed ABS models of GPS tags to test methods of attachment to Red Knots (*Calidirs canutus*). This allowed the authors to test attachment methods without the risk of losing expensive GPS tags. While traditional methods can be used for mock‐ups, 3D printing uses less material (due to the additive method, Gardner & Olson, 2016) and can produce accurate replicas quickly and easily.

### 4.2 Limitations

There are limitations of using 3D printed models for research due to their static nature. These problems are no different to the limitations experienced by models made using traditional manufacturing techniques. Some of these problems could be overcome by employing 3D printed shells into which robotics could be mounted. Robots are beginning to be used in studies of animal behavior (see Frohnwieser, Murray, Pike, & Wilkinson, 2016 for a review), and this simple adaptation may yield interesting results. Using models in a manipulative experimental approach allows greater control over specific elements such as size of ornamentation, coloration, movement, or removal of olfactory cues (e.g., Heinen‐Kay et al., 2015), but this is at the potential cost of realism. Therefore, a balance must be struck between absolute realism (which can only be achieved by using an organism), and manipulative control over specific elements important for the study. 3D printing can be of great benefit to such studies allowing models to be created with differences which would not be physically or ethically possible with live animals. There are, though, caveats to using models, including the potential that other factors are involved, for example signals, which are not manipulated through the use of models. As such, the observed responses may not be as "true" as when using an organism. However, by the same token, this method does provide the ability to precisely change only one element, which cannot be guaranteed with treatments or manipulations involving organisms. Observational or correlational studies can be used to address similar questions but rely only on natural variation. Observational studies may require more time and can suffer from confounding variables or reverse causation but do allow for testing in a biologically meaningful manner which might be lacking in the laboratory. For observational studies, 3D printing can be used to produce equipment. One practical limitation of 3D printing is the size of the object which can be printed. Most 3D printers have a relatively small print size (see Table 2). Objects larger than this print size can be made by incorporating joining features (e.g., sockets and pins) into the 3D prints or include positions for traditional fasteners (e.g., screws or nuts and bolts) to be used. These features can be added into the CAD drawings of parts, and some software allow for the inclusion of threaded holes to make assembly easier and faster. Large 3D prints often have lower resolution due to limitations of current scanning and printing methods, while smaller models can be printed at higher resolution but may be more expensive as a result.

Despite the advantages of 3D printing, many of the materials used in this process have not undergone testing for durability or toxicology. Where testing has occurred it often indicates leaching of chemicals from the materials. In one example, leached chemicals had a negative impact on the growth of zebrafish (*Danio rerio*) embryos (Oskui et al., 2016). It seems prudent to suggest that materials likely to be in contact with living organisms need to be well researched first, given the release of chemicals from the 3D print may affect an animal's behavior or even lead to death. Additional concerns center on environmental impact, and while some feedstock used in 3D printing is recyclable (such as thermoplastic polymers used in FFD 3D printing, Table 1), others such as photopolymers (used in SLA 3D printing) are not. There are, however, continual developments, for example, the toxicology of some models can be reduced with increased ultraviolet light treatment (Oskui et al., 2016) and new printable plastics that are recyclable are in development (Mohammed, 2016). Additional problems with 3D printed equipment in the laboratory include the lack of guarantee and possibility of a short lifespan, but if costs are low, this can be solved by reprinting the object. With sensible precautions, 3D printing can make many beneficial changes to the way we produce objects.

## 5 TAKE HOME MESSAGES

1. 3D printing enables the rapid production of items for low unit cost by commercial suppliers, or if many experimental pieces are to be printed then this can be done in‐house. Printed items can be models of delicate bones, complex biological structures, microfluidic chambers, labware, or even hypothetical ancestral structures. 3D printed models can remove the reliance on the use of museum‐preserved specimens.
2. A major limitation to the adoption of 3D printing is the initial cost of the printer, but this is falling. The cost of CT or laser scanning any structure to be modeled can also be high, but with suitable CAD software, many items can be approximated to a reasonable degree and custom items designed.
3. The impact of 3D printing on the environment is only just being studied. Many materials are not recyclable, and some plastics may release chemicals into the environment. The amount of chemicals released and the effects these may have is unknown.
4. Sharing of 3D models online is creating a large repository of objects that can be downloaded and printed, allowing anyone with a 3D printer to produce them. This can facilitate replication of experiments in a way never before possible.
5. The overarching advantage of 3D printing is the freedom given to researchers, allowing them to print custom objects, quickly and at relatively low cost. The application of 3D printing in Ecology and Evolution has begun but the technique offers many more opportunities for the future.

## AUTHOR CONTRIBUTION

MW designed, researched, and wrote the manuscript. SH aided with writing.

## Data Availability

There are no novel data presented in this manuscript.
